# Metabolic inhibition reduces cardiac L-type Ca^2+^ channel current due to acidification caused by ATP hydrolysis

**DOI:** 10.1371/journal.pone.0184246

**Published:** 2017-08-31

**Authors:** Giedrius Kanaporis, Rimantas Treinys, Rodolphe Fischmeister, Jonas Jurevičius

**Affiliations:** 1 Institute of Cardiology, Lithuanian University of Health Sciences, Kaunas, Lithuania; 2 INSERM UMR-S 1180, Univ Paris-Sud, Université Paris-Saclay, Châtenay-Malabry, France; Cinvestav-IPN, MEXICO

## Abstract

Metabolic stress evoked by myocardial ischemia leads to impairment of cardiac excitation and contractility. We studied the mechanisms by which metabolic inhibition affects the activity of L-type Ca^2+^ channels (LTCCs) in frog ventricular myocytes. Metabolic inhibition induced by the protonophore FCCP (as well as by 2,4- dinitrophenol, sodium azide or antimycin A) resulted in a dose-dependent reduction of LTCC current (I_Ca,L_) which was more pronounced during β-adrenergic stimulation with isoprenaline. I_Ca,L_ was still reduced by metabolic inhibition even in the presence of 3 mM intracellular ATP, or when the cell was dialysed with cAMP or ATP-γ-S to induce irreversible thiophosphorylation of LTCCs, indicating that reduction in I_Ca,L_ is not due to ATP depletion and/or reduced phosphorylation of the channels. However, the effect of metabolic inhibition on I_Ca,L_ was strongly attenuated when the mitochondrial F_1_F_0_-ATP-synthase was blocked by oligomycin or when the cells were dialysed with the non-hydrolysable ATP analogue AMP-PCP. Moreover, increasing the intracellular pH buffering capacity or intracellular dialysis of the myocytes with an alkaline solution strongly attenuated the inhibitory effect of FCCP on I_Ca,L_. Thus, our data demonstrate that metabolic inhibition leads to excessive ATP hydrolysis by the mitochondrial F_1_F_0_-ATP-synthase operating in the reverse mode and this results in intracellular acidosis causing the suppression of I_Ca,L_. Limiting ATP break-down by F_1_F_0_-ATP-synthase and the consecutive development of intracellular acidosis might thus represent a potential therapeutic approach for maintaining a normal cardiac function during ischemia.

## Introduction

Mitochondria play a central role in cellular energy production and Ca^2+^ homeostasis. During myocardial ischemia, mitochondria undergo progressive damage, including dramatic decrease in the activity of oxidative-phosphorylation complexes [1]. The resulting metabolic stress disrupts intracellular Ca^2+^ cycling via alterations in sarcoplasmic reticulum (SR) Ca^2+^ load [2–5], causing an impairment of excitation-contraction coupling (ECC) and cardiac contractility [6, 7]. In addition, ischemia leads to heterogeneities in cardiac excitability and refractoriness and creates a substrate for ectopic excitation that can trigger lethal ventricular arrhythmias [8, 9].

L-type Ca^2+^ channel current triggers the release of Ca^2+^ from the SR. Alterations in the density or function of LTCCs have been implicated in a variety of cardiovascular diseases, including atrial fibrillation [10, 11], heart failure [12] and ischemic heart disease [13]. While several studies showed that metabolic inhibition as occurs during ischemia induces a decrease in the amplitude of I_Ca,L_ [14, 15], the underlying mechanisms remain poorly understood. Metabolic inhibition results in multiple and complex changes in cardiomyocytes such as fall of ATP levels [16], elevation in inorganic phosphate, ADP, free Mg^2+^ [3, 17] and Ca^2+^ [18] concentrations and intracellular acidification [3, 19, 20], all of which could affect the activity of LTCCs. In addition, a close proximity or even tethering of mitochondria to endo/sarcoplasmic reticulum and plasma membrane has been documented in several cell types [21, 22]. These organelles may therefore form restricted domains with elevated changes in metabolite and ion concentrations that could intensify the effects on the activity of ion channels.

The aim of this study was to investigate the mechanisms by which metabolic inhibition causes a reduction in I_Ca,L_. The experiments were performed in frog cardiomyocytes rather than mammalian cardiomyocytes, because in these cells LTCCs are the primary source of Ca^2+^ for contraction and Ca^2+^ release from the SR plays only a minor role [23, 24]. We found that during metabolic inhibition the amplitude of both basal and stimulated I_Ca,L_ was significantly attenuated. This effect was at least partially reversed by the inhibition of mitochondrial F_1_F_0_-ATP synthase or intracellular dialysis with non-hydrolysable ATP analogues. We demonstrate that metabolic inhibition effect is dependent on the intracellular acidification of the cardiomyocytes presumably resulting from the reverse-mode activity of F_1_F_0_-ATP synthase.

## Materials and methods

### Animals and myocyte isolation

All procedures were approved by the State Food and Veterinary Service of the Republic of Lithuania and comply with the Guide for the Care and Use of Laboratory Animals of the National Institutes of Health and UK regulations on animal experimentation [25]. Ventricular cells were enzymatically dispersed from frog (*Rana esculenta*, 18 frogs in total) heart. Frogs were purchased from the Etablissements Guy Couétard (La Fradinière, Saint Hilaire de Riez, France) where the animals were bred for research purposes. For the preparation of frog ventricular myocytes, the ionic composition of Ca^2+^-free Ringer solution was (in mM): 88.4 NaCl; 2.5 KCl; 23.8 NaHCO_3_; 0.6 NaH_2_PO_4_; 1.8 MgCl_2_; 5 creatine; 10 *D*-glucose; pH 7.4. Frogs were euthanized by concussion. The heart was removed and submersed in cold Ca^2+^-free Ringer solution (4–8°C) supplemented with 80 μM CaCl_2_. Then the heart was mounted on a Langendorff apparatus and perfused at 5 ml/min at 30°C with a series of solutions based on a Ca^2+^-free Ringer solution. At first, the heart was perfused for 8–10 min with Ca^2+^-free Ringer solution containing 20 μM of EGTA and 1 mg/ml fatty acid-free bovine serum albumin (BSA). After that, perfusion was switched to the isolation solution composed of Ca^2+^-free Ringer solution with 0.2 mg/ml of trypsin type XIII (Sigma-Aldrich, Schnelldorf, Germany), 0.14 mg/ml of collagenase (Yakult S, Tokyo, Japan) and 1 mg/ml of BSA added. Perfusion lasted for 10–12 min at 30°C. The solution was then changed to a fresh enzymatic solution, and the heart was perfused for another 10–12 min period. Finally, the ventricle was dissected and the cells were dispersed in a flask containing a Ca^2+^-free Ringer solution supplemented with 80 μM CaCl_2_. Isolated cells were stored at 4°C in storage Ringer solution composed of Ca^2+^-free Ringer solution supplemented with: 1 mM CaCl_2_, 10 μl/ml non-essential and essential amino acid and vitamin solution (MEM 100x), and antibiotics (200 u/ml penicillin and 0.2 mg/ml streptomycin).

### Electrophysiology

A few drops of cell suspension were placed in a perfusion chamber mounted on an inverted microscope stage. After the cells had settled to the bottom, the chamber was superfused with K^+^ free control external solution containing (in mM): 107 NaCl;10 HEPES; 20 CsCl; 4 NaHCO_3_; 0.8 NaH_2_PO_4_; 1.8 MgCl_2_; 1.8 CaCl_2_; 5 *D*-glucose; 5 sodium pyruvate; pH 7.4 was adjusted with NaOH. Patch pipettes were made from glass capillaries (Drummond, Broomall, PA, USA) and had resistances of 0.7–1.2 MΩ when filled with control internal solution. The myocytes were dialysed with control internal solution composed of (in mM): 120 CsCl, 5 EGTA (acid form), 4 MgCl_2_, 0.062 CaCl_2_, 5 creatine phosphate disodium salt, 3 Na_2_ATP, 0.42 Na_2_GTP and 10 HEPES; pH 7.3 was adjusted with CsOH. External control or drug-containing solutions were applied to the myocyte by placing the cell at the opening of capillary tubing (800 μm in inner diameter) flowing at a rate of ≈150 μl/min. Changes in extracellular solutions were automatically achieved by a rapid solution changer (RSC100, Bio-Logic, France). I_Ca,L_ was recorded under the whole-cell configuration of the patch-clamp technique. I_Ca,L_ was activated every 8 s by depolarizing voltage clamp steps from –80 mV holding potential to 0 mV for 200 ms. I_Ca,L_ amplitude was measured as the difference between peak inward current and the current at the end of the 200 ms pulse. Application of tetrodotoxin (3 μM) was used to inactivate voltage-dependent Na^+^ currents. K^+^ currents were blocked by replacing all intracellular and extracellular K^+^ ions with Cs^+^. The cells were voltage-clamped using a patch-clamp amplifier RK-400 (Bio-Logic, Claix, France). Currents were sampled at a frequency of 10 kHz using a 16-bit analogue-to-digital converter (PCL816, Advantech France, Levallois-Perret, France). Liquid junction potentials between internal and external solutions were compensated before pipette touched the cell. Currents were not compensated for capacitive and leak currents.

**Fig 1:**
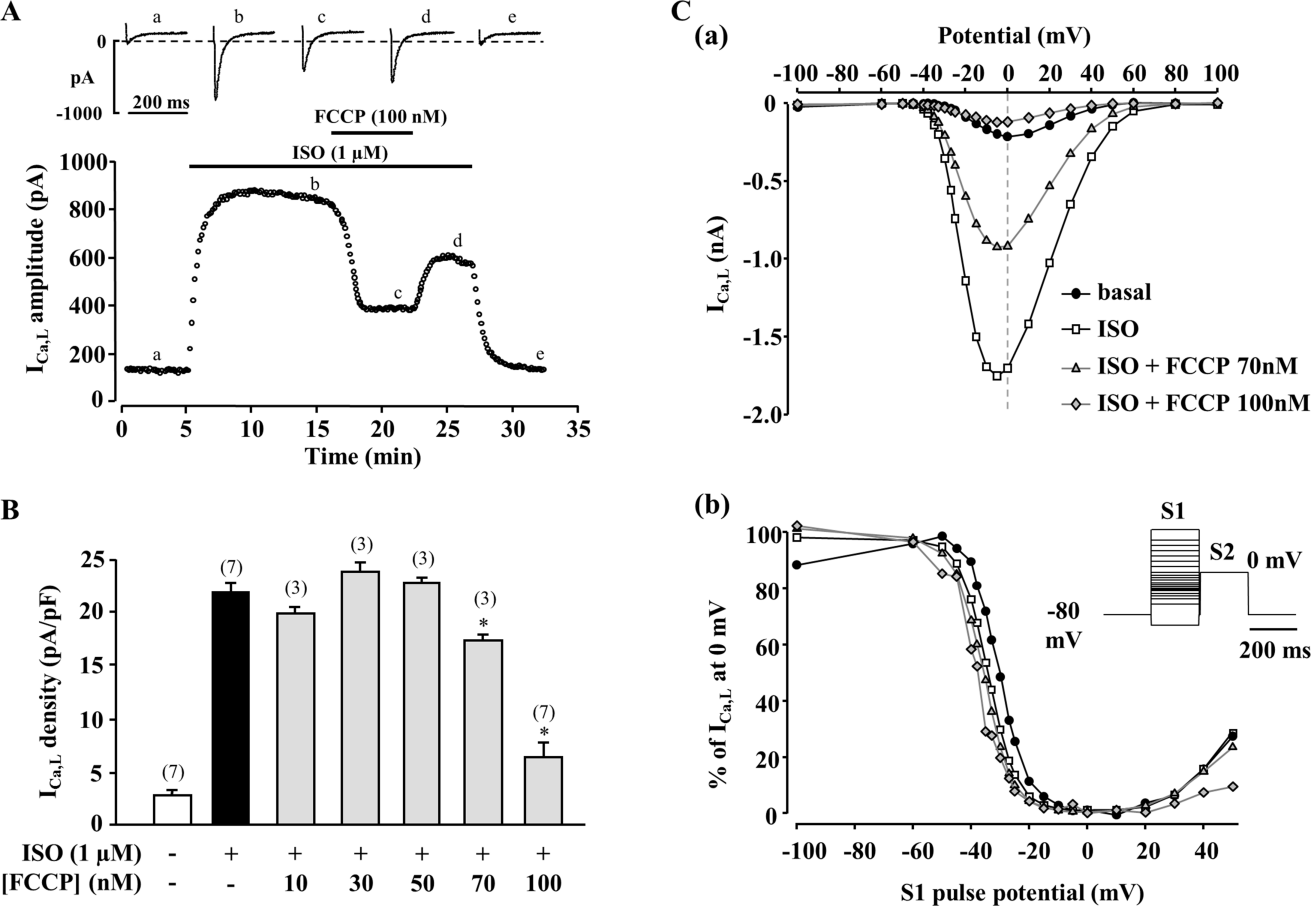
Effect of FCCP on isoprenaline stimulated I_Ca,L_. (A) Effect of FCCP on isoprenaline (ISO, 1 μM)-stimulated I_Ca,L_. Traces of I_Ca,L_ shown on the top were recorded at the times indicated by the corresponding letters on the main graph. (B) Concentration-response curve for the effect of FCCP on ISO-stimulated I_Ca,L_. (C) Current-voltage (I-V) relationships (a) and inactivation curves (b) of I_Ca,L_ under basal conditions, during ISO (1 μM) stimulation and during exposure to two different FCCP concentrations (70 and 100 nM) in the presence of ISO. Inset: Double-pulse protocol used for the inactivation curves (see Methods for details). I_Ca,L_ peak amplitude during S2 pulse is expressed as a percentage of I_Ca,L_ measured at 0 mV without conditioning pulse S1 and plotted as a function of S1 pulse potential.

To determine current-voltage (I-V) relationship and inactivation of I_Ca,L_, a double pulse voltage-clamp protocol was applied every 4 seconds (see insert in Fig 1C). During the first pulse S1, the membrane potential was set at membrane potentials ranging from -100 to *+*100 mV for 200 ms. S1 pulse was followed by a 3 ms repolarization to the -80 mV holding potential and then a depolarizing pulse S2 to 0 mV was applied for 200 ms.

All experiments were performed at room temperature (18–24°C), and the temperature did not change by more than 1°C during an experiment.

### Drugs and reagents

Tetrodotoxin was from Latoxan (Rosans, France). All other drugs were purchased from Sigma-Aldrich (Schnelldorf, Germany) unless otherwise indicated. Each day, fresh 1–10 mM stock solutions were prepared and stored at 4°C. FCCP (p-trifluoromethoxy carbonyl cyanide phenyl hydrazone) was dissolved in ethanol, DNP (2,4-dinitrophenol) and antimycin A in DMSO. The stock solution of oligomycin was prepared in ethanol. To improve its solubility, the control external solution was heated to 50°C and appropriate amounts of stock solution of oligomycin were then added. All other drugs were dissolved in water based solutions. When the stock solutions were obtained in DMSO or ethanol, the corresponding amount of solvent was also added to the control external solution.

### Data and statistics analysis

Error bars are given as standard errors to the mean (S.E.M.). The number of cells is denoted by n. For statistical evaluation the paired and unpaired Student’s *t*-test were used, and a difference was considered statistically significant with P<0.05. In the text, “basal I_Ca,L_” refers to the Ca^2+^ current which was not stimulated by β-adrenergic stimulation or intracellular cAMP.

## Results

### Effect of metabolic inhibitors on I_Ca,L_

**Fig 2:**
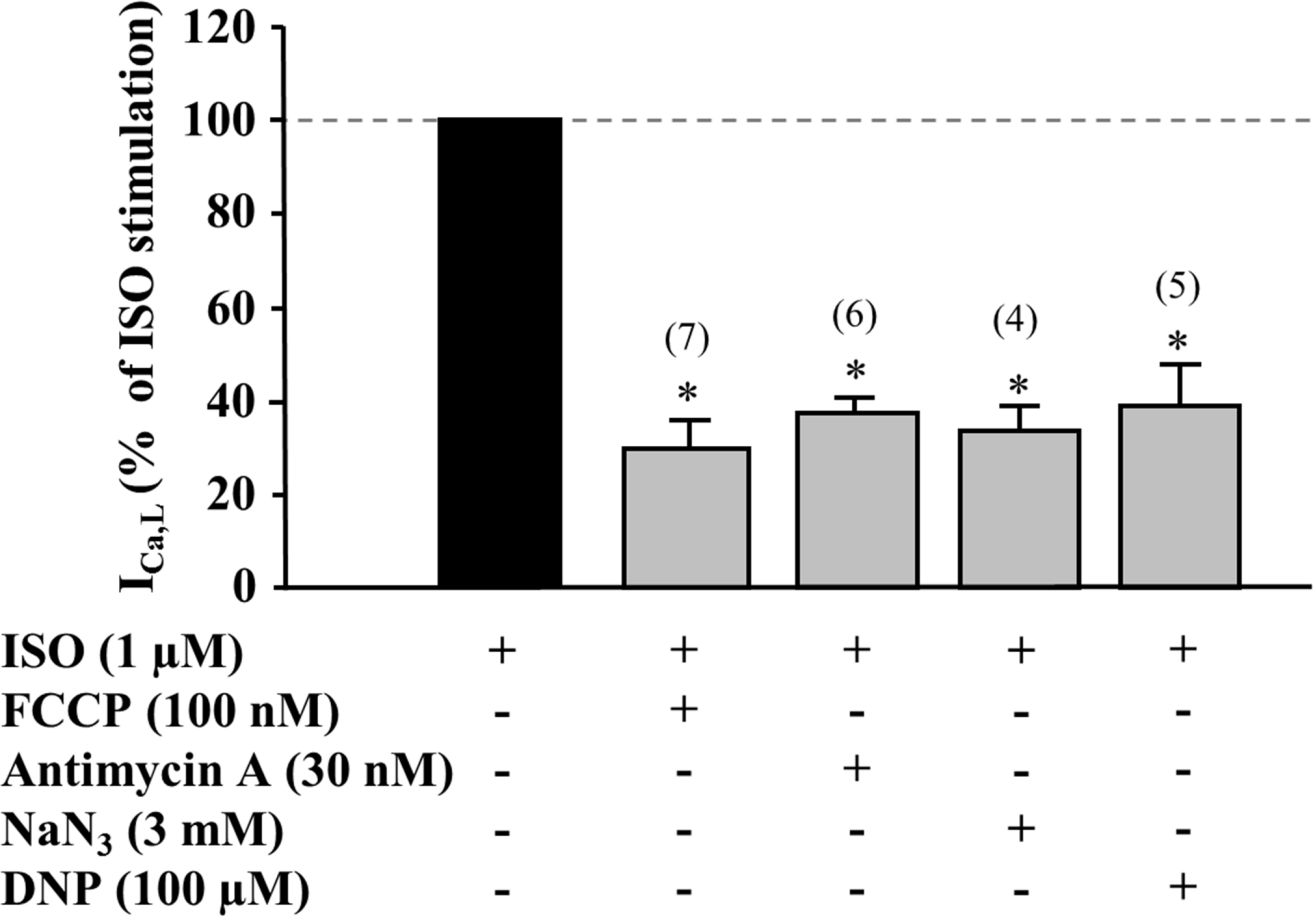
Effect of various metabolic inhibitors on isoprenaline stimulated I_Ca,L_. Peak amplitude of ISO-stimulated I_Ca,L_ during exposure of the cells to various inhibitors of oxidative phosphorylation. Values are presented as means ± SEM for the number of cells indicated in parentheses. * P<0.05 *vs*. ISO alone.

The first series of experiments were set to determine the effect of metabolic inhibition on I_Ca,L_ in frog ventricular myocytes. The mitochondrial uncoupler carbonyl cyanide p- [trifluoromethoxy]-phenyl-hydrazone (FCCP) had no significant effect on basal I_Ca,L_ at 30 to 70 nM but reduced the current at 100 nM concentration by 22.1 ± 9.6% (n = 5, P<0.05, Supplemental S1 Fig). When I_Ca,L_ was enhanced by the β-adrenergic agonist isoprenaline (ISO, 1 μM), the inhibitory effect of FCCP was more pronounced, and was already significant at 70 nM. At 100 nM concentration, FCCP suppressed the ISO-stimulated I_Ca,L_ by 70.4 ± 6.6% (n = 7, P<0.05) (Fig 1A and 1B). Application of FCCP had no major effect on the shape of the I_Ca,L_ I-V relationship (Fig 1Ca) or inactivation curve (Fig 1Cb).To examine whether the inhibitory effect of FCCP on I_Ca,L_ was due to inhibition of oxidative phosphorylation, other metabolic inhibitors were tested. 2,4- dinitrophenol (DNP, 100 μM), another uncoupler of oxidative phosphorylation, caused a similar reduction of ISO-stimulated I_Ca,L_ as 100 nM FCCP (60.9 ± 8.7% inhibition, n = 5, P<0.05) (Fig 2). Sodium azide (NaN_3_, 3 mM), an inhibitor of respiratory chain complex IV, or antimycin A (30 nM), an inhibitor of complex III, decreased basal I_Ca,L_ by 46.6 ± 13.6% (n = 3, P<0.05) and 71.9 ± 2.4% (n = 4, P<0.01), and ISO-stimulated I_Ca,L_ by 66.3 ± 5.4% (n = 4, P<0.05) and 62.5 ± 2.9% (n = 6, P<0.05), respectively (Fig 2 and experimental traces are shown in supplemental S2 Fig). These results demonstrate that various metabolic inhibitors, with different structures and targets cause a decrease of basal and stimulated I_Ca,L_, indicating that inhibition of oxidative phosphorylation suppresses the activity of LTCCs.

### Effect of intracellular ATP on I_Ca,L_

**Fig 3:**
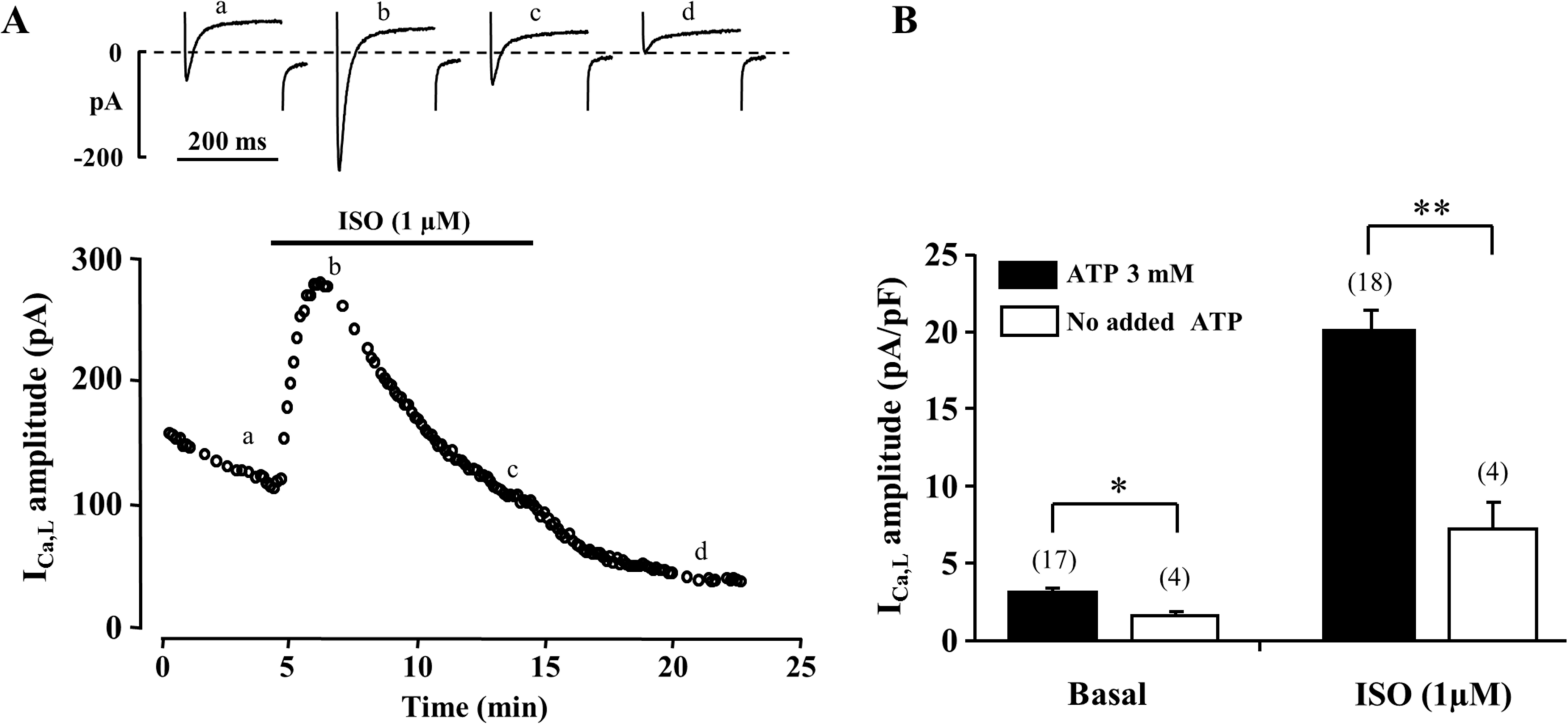
Effect of intracellular ATP on I_Ca,L_ amplitude. (A) A typical experiment representing the effect of ISO (1 μM) on basal I_Ca,L_ in the absence of ATP in pipette solution. The current traces shown on the top of the panel were recorded at the times indicated by the corresponding letters on the main graph. (B) Mean basal and ISO-stimulated I_Ca,L_ amplitudes recorded in cells dialyzed with 3 mM ATP and ATP-free internal solutions. *P<0.05, **P<0.01 for the number of cells indicated in parentheses.

Inhibitors of cellular metabolism are expected to disturb ATP synthesis and therefore could lead to suppression of I_Ca,L_ due to insufficient intracellular ATP levels. Therefore, we evaluated the significance of the presence of ATP in the pipette solution for the maintenance of I_Ca,L._ In control conditions, when cells were dialyzed with a solution containing 3 mM ATP, the mean basal I_Ca,L_ amplitude was 3.1 ± 0.3 pA/pF (n = 17). In the presence of ISO (1 μM), I_Ca,L_ increased ~7-fold to 20.0 ± 1.3 pA/pF (n = 18). When ATP was excluded from the internal solution (Fig 3) the mean basal I_Ca,L_ amplitude was much lower (1.6 ± 0.1 pA/pF, n = 4) and increased only by ~4-fold to 7.3 ± 1.7 pA/pF in the presence of ISO (n = 4) (Fig 3B). Additionally, the lack of ATP in the internal solution also accelerated the spontaneous run-down of I_Ca,L_ (Fig 3A).

### Role of ATP-synthase in the effect of metabolic inhibition on I_Ca,L_

**Fig 4:**
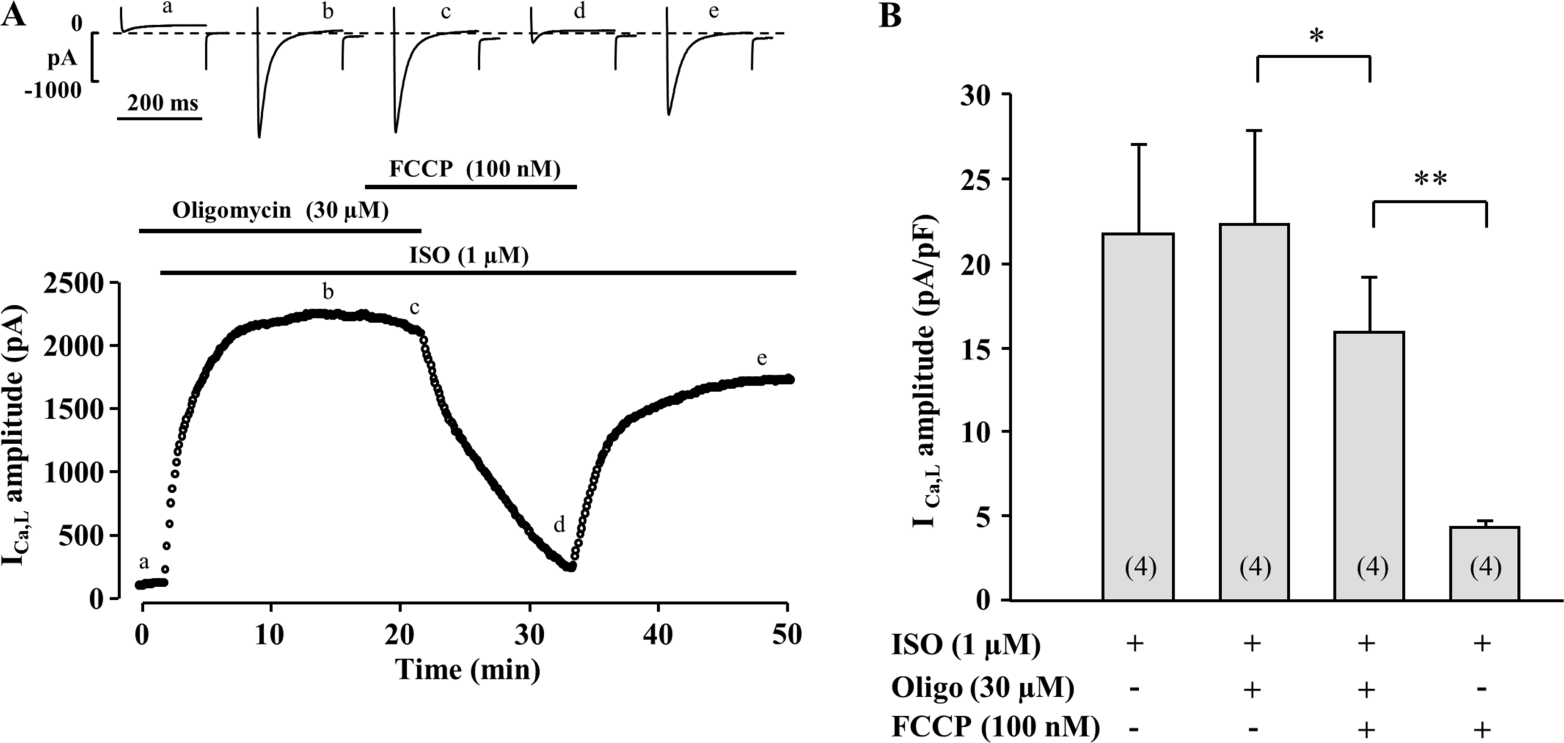
Effect of oligomycin on I_Ca,L_ and its response to FCCP. (A) A typical experiment showing the effect of a pretreatment of the cell with oligomycin (30 μM) on ISO (1 μM)-stimulated I_Ca,L_ and on the response to FCCP. (B) Mean amplitude of ISO-stimulated I_Ca,L_ recorded in the absence and presence of FCCP (100 nM) and oligomycin (30 μM). *P<0.05, **P<0.005.

The obvious explanation for the results obtained so far is that LTCCs require ATP to function properly and metabolic inhibition reduces ATP level because oxidative phosphorylation is stopped. However, one caveat to this hypothesis is that the inhibitory effects on I_Ca,L_ of all four metabolic inhibitors were observed even though 3 mM ATP was present in the patch pipette solution throughout the entire experiment. One would thus expect this permanent exogenous supply of ATP to minimize the role of endogenous ATP-synthase activity. To specifically evaluate the contribution of this enzyme in the regulation of I_Ca,L_, we examined the effect of oligomycin, an F_1_F_0_-ATP-synthase inhibitor. Oligomycin (30 μM) had no effect on either basal (not shown) or ISO stimulated I_Ca,L_ (Fig 4). These results indicate that mitochondrial ATP production is not necessary for the activity of LTCCs, as long as ATP is supplied to the cell via the patch pipette. However, although oligomycin had no significant effect on I_Ca,L_ on its own, oligomycin strongly blunted the effect of FCCP (Fig 4A and 4B). In the same cells, washout of oligomycin recovered a strong inhibitory effect of FCCP on I_Ca,L_. Thus, oligomycin strongly antagonized the inhibitory effect of FCCP on I_Ca,L_. These results indicate that the activity of mitochondrial F_1_F_0_-ATPase contributes to the decrease in I_Ca,L_ during metabolic inhibition. Under normal conditions, the F_1_F_0_-ATPase complex is the site of mitochondrial ATP production. However, in the presence of a metabolic inhibitor such as FCCP electrical potential across the inner mitochondrial membrane is lost and the F_1_F_0_-ATPase complex may run backwards and consume ATP [26–28]. This reverse activity may thus cause a local ATP depletion that leads to a reduction in I_Ca,L_.

### Role of ATP depletion

**Fig 5:**
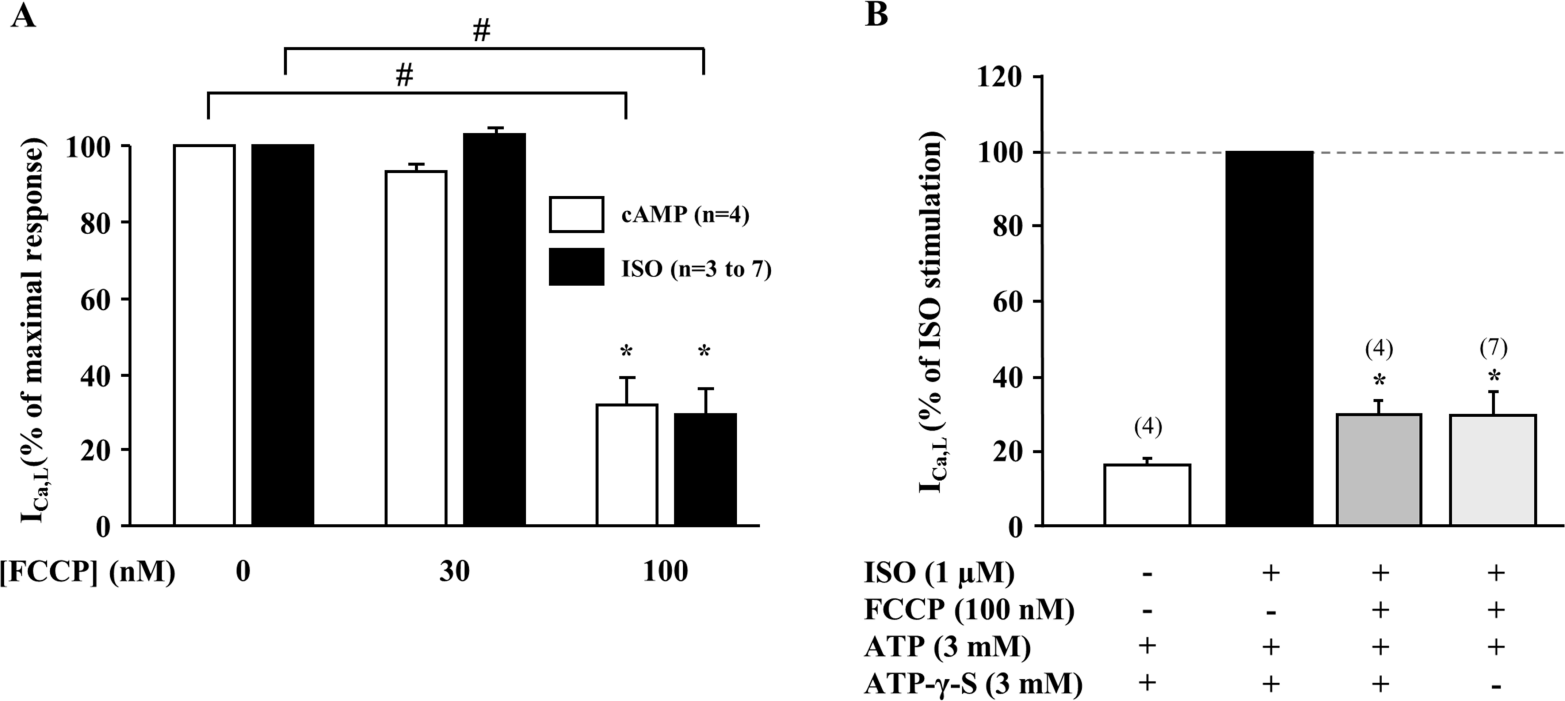
Role of channel phosphorylation in the effect of FCCP on I_Ca,L_. (A) Comparison of the effect of a metabolic inhibition induced by FCCP on I_Ca,L_ stimulated by either ISO (1 μM) or intracellular cAMP (20 μM). (B) Comparison of the effect of FCCP on ISO-stimulated I_Ca,L_ in the presence or absence of ATP-γ-S (3 mM) in the pipette-filling solution. * P<0.05 and # p<0.05 *vs* absence of FCCP.

The effect of ATP depletion is expected to be more prominent during β-adrenergic stimulation, since ATP is required for cAMP production by adenylyl cyclase, and for phosphorylation of the LTCCs. To test this hypothesis, two series of experiments were performed. First, we tested the effect of FCCP in cells in which I_Ca,L_ was stimulated by direct intracellular application of cAMP (20 μM) via the patch pipette, thus bypassing the activation β-adrenergic receptor and adenylyl cyclase. Dialysis of the cell with cAMP stimulated I_Ca,L_ to 21.5 ± 4.2 pA/pF (n = 4) and the amplitude of the current was not significantly different from that obtained with 1 μM ISO. Fig 5A shows that FCCP still produced a dose-dependent inhibition of I_Ca,L_ stimulated by cAMP, and the effect was not significantly different from the one observed when the current was stimulated by ISO. Thus, the suppression of I_Ca,L_ during metabolic inhibition does not involve a reduction in cAMP production due to lack of ATP, but rather involves mechanisms located downstream from β-adrenergic receptor and adenylyl cyclase. In the second series of experiments, to test whether metabolic inhibition induced reduction in I_Ca,L_ is determined by insufficient phosphorylation of LTCCs, the patch pipette solution containing 3mM ATP was supplemented with the hydrolysis-resistant analogue of ATP, adenosine 5′-O-(3-thiotriphosphate) (ATP-γ-S, 3 mM), which leads to irreversible thiophosphorylation of proteins [29]. Irreversible phosphorylation of LTCCs was confirmed by the observation that under these conditions ISO induced increase of I_Ca,L_ was maintained even after the prolonged washout of ISO (S3 and S4 Figs). If during metabolic inhibition the decrease in I_Ca,L_ is caused by the reduced phosphorylation of LTCCs, then FCCP is expected to have no effect on I_Ca,L_ after irreversible thiophosphorylation of the channels. However, as shown in Fig 5B, application of FCCP (100 nM) decreased I_Ca,L_ stimulated with ISO and ATP-γ-S by 70.6 ± 4.1% (n = 4), an effect which was not different from when the cells were dialyzed with ATP alone. Experimental traces demonstrating the effect of FCCP in cells dialyzed with ATP-γ-S are shown in supplemental S3 Fig. These results indicate that protein dephosphorylation is not the cause of I_Ca,L_ reduction during metabolic inhibition.

### Role of ATP hydrolysis

**Fig 6:**
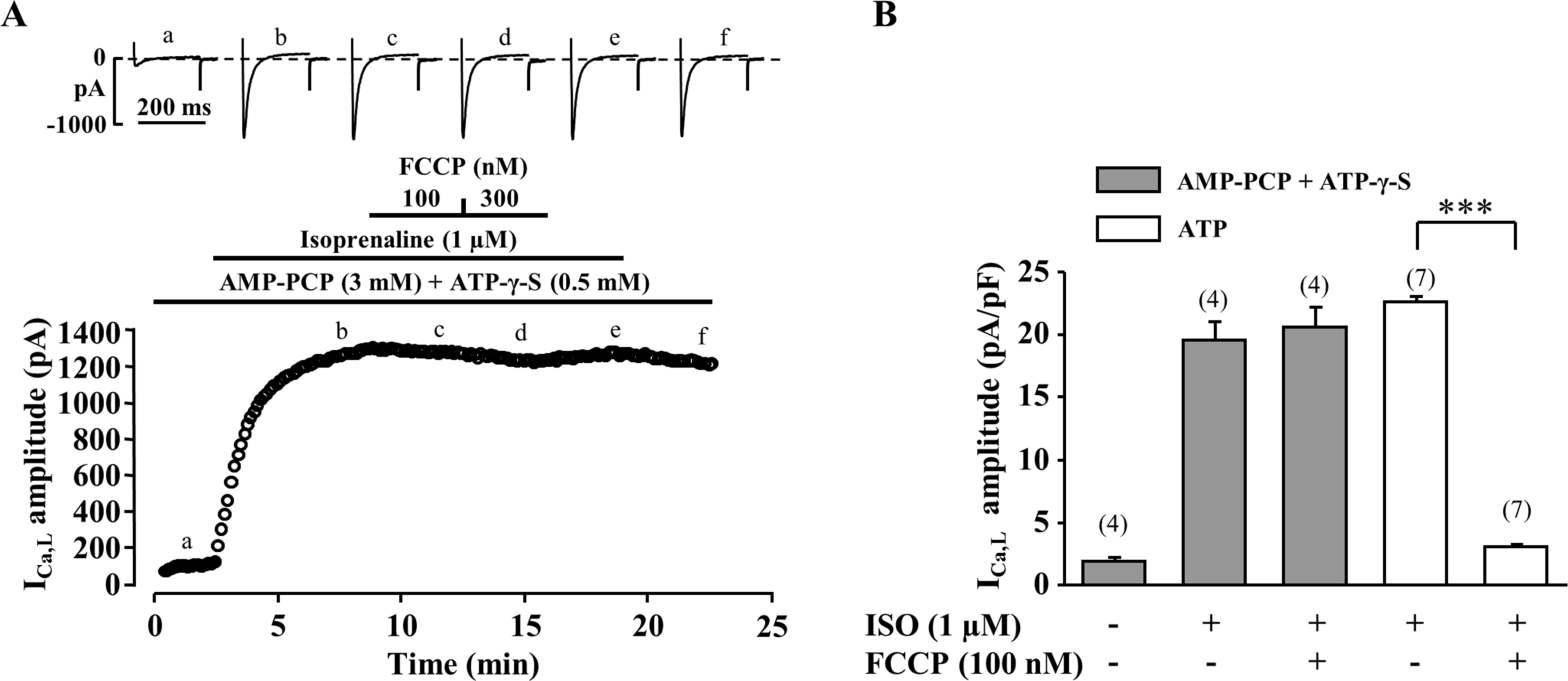
Non-hydrolysable ATP analogues prevent decrease of I_Ca,L_ by metabolic inhibition. (A) A typical experiment showing the absence of effect of FCCP (100 and 300 nM) in a myocyte dialyzed with a pipette solution in which ATP was substituted with non-hydrolysable ATP analogues AMP-PCP (3 mM) and ATP-γ-S (0.5 mM). (B) Mean amplitudes of I_Ca,L_ recorded in control and with ISO (1 μM) during metabolic inhibition in cells dialyzed with 3 mM of ATP (white bars) or with non-hydrolysable ATP analogues (grey bars). *** P<0.001.

So far, our results demonstrate that the regulation of I_Ca,L_ by metabolic inhibition involves the F_1_F_0_-ATPase complex but not the subsequent reduction in ATP. Therefore, we hypothesized that ATP hydrolysis is necessary in this process, possibly through changes in the concentration of one or several metabolites. To test this hypothesis, we examined whether FCCP still reduced I_Ca,L_ in cells dialysed with a solution containing only non-hydrolysable ATP analogues. In the experiment shown in Fig 6A, a frog ventricular cell was dialyzed with a pipette solution where ATP was replaced by a combination of 5'-adenylyl (β,γ-methylene) diphosphonate (AMP-PCP, 3 mM) and ATP-γ-S (0.5 mM). ATP-γ-S was added to the solution only to allow for the stimulation of I_Ca,L_ by PKA upon application of ISO. As shown, inhibition of I_Ca,L_ by FCCP was completely abolished under these conditions. Even a 3-fold larger concentration of FCCP (300 nM, n = 4, Fig 6A), or application of antimycin A (30 μM, supplemental S4 Fig), failed to suppress I_Ca,L_. Therefore, we conclude that ATP hydrolysis is required for the inhibition of I_Ca,L_ during metabolic inhibition.

### Role of Mg^2+^

**Fig 7:**
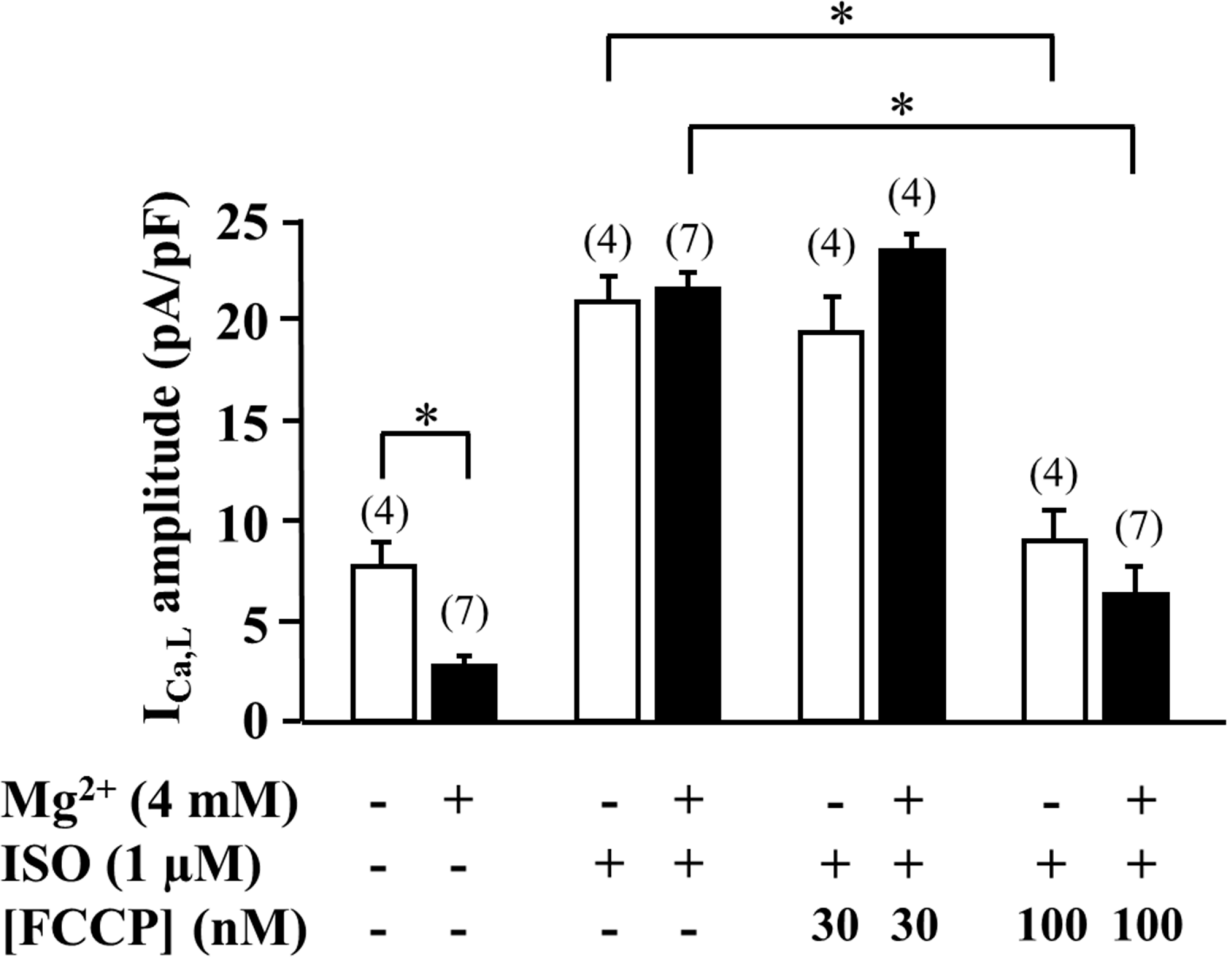
Effect of FCCP on I_Ca,L_ in cells dialyzed with zero-Mg^2+^ solution. Comparison of the response to ISO (1 μM) and to FCCP (30 and 100 nM) in the presence of ISO in the absence (white bars) or presence (black bars) of 4 mM Mg^2+^ in the pipette solution. *P<0.05.

As ATP has a higher affinity for Mg^2+^ than ADP, ATP hydrolysis during metabolic inhibition can lead to a rise in cytosolic Mg^2+^ concentration ([Mg^2+^]_i_) [17] and [Mg^2+^]_i_ has been shown to reduce the activity of LTCCs [30–32]. To test for a possible role of [Mg^2+^]_i_ changes in the effects of metabolic inhibitors on I_Ca,L_, Mg^2+^ in the pipette solution was substituted with Na^+^. The absence of Mg^2+^ in the internal solution led to an increase in basal I_Ca,L_ (7.7 ± 1.2 pA/pF (n = 4) *vs*. 3.1 ± 0.4 pA/pF (n = 7) in control), but no significant effect on the ISO-stimulated I_Ca,L_ was observed. In addition, FCCP (100 nM) still inhibited I_Ca,L_ in Mg^2+^-free solution similarly to when Mg^2+^ (4 mM) was present in the patch pipette solution (Fig 7). These results indicate that the effect of metabolic inhibition on I_Ca,L_ is not mediated by changes in [Mg^2+^]_i_.

### Role of intracellular acidosis

**Fig 8:**
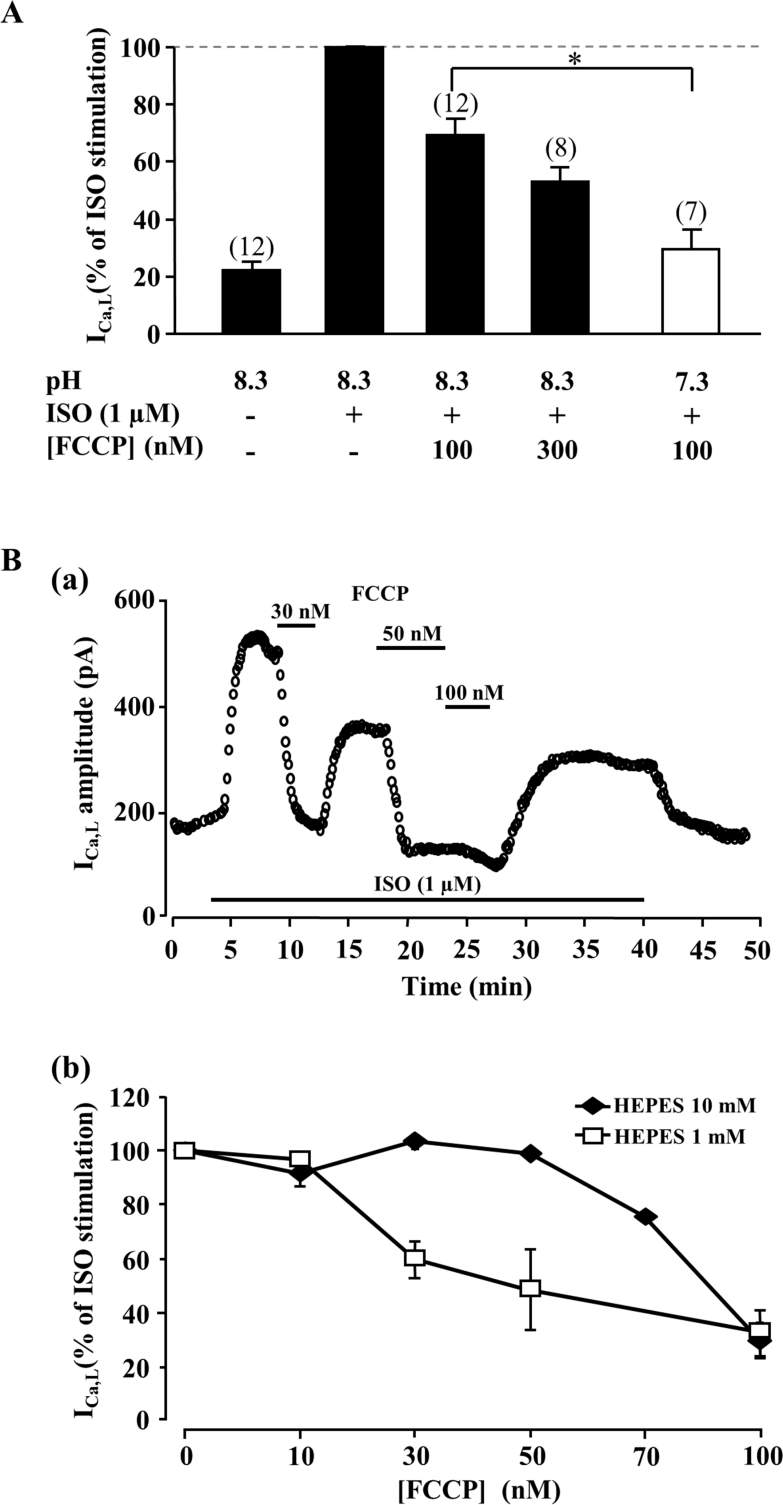
Effect of intracellular pH on I_Ca,L_ response to metabolic inhibition. (A) Effect of 100 and 300 nM of FCCP on ISO (1 μM)-stimulated I_Ca,L_ in cells dialyzed with either control (pH 7.3, white bar) or alkaline solution (pH 8.3, black bars). * P<0.05. (B) (a) Effect of three different FCCP concentrations (30, 50 and 100 nM) on ISO (1 μM)-stimulated I_Ca,L_ in a ventricular myocyte dialysed with low HEPES (1 mM) intracellular solution. (b) Concentration-response curves for the effects of FCCP on ISO-stimulated I_Ca,L_ in cells dialysed with a solution containing either 1 mM (n = 3 to 7, white squares) or 10 mM HEPES (n = 4, black diamonds).

Development of intracellular acidosis during metabolic inhibition in cardiomyocytes is well documented [3, 19, 20]. In order to investigate the contribution of acidosis in the effect of metabolic inhibition on I_Ca,L_, myocytes were dialyzed with alkaline (pH 8.3) internal solution and the effect of FCCP on I_Ca,L_ was tested. Dialysis of myocytes with alkaline solution strongly attenuated the inhibitory effect of FCCP on I_Ca,L_. As shown in Fig 8A, 300 nM FCCP caused a significantly smaller decrease in I_Ca,L_ than 100 nM FCCP in cells dialysed with control solution with pH 7.3.

To further examine the role of cytosolic acidification in the inhibitory effect of metabolic inhibition on I_Ca,L_, the effect of FCCP was examined in cells dialysed with an internal solution with a low pH buffer concentration (1 mM HEPES instead of 10 mM in control conditions). As expected, reducing the pH buffering capacity in the pipette solution amplified the inhibitory effect of FCCP on I_Ca,L_ as evidenced by a shift to lower FCCP concentrations of the concentration-response curve for the effect of FCCP on ISO-stimulated I_Ca,L_ (Fig 8B). Thus, we conclude that the main cause for I_Ca,L_ inhibition in frog ventricular myocytes during metabolic inhibition is due to ATP hydrolysis causing an intracellular acidosis in the vicinity of LTCCs.

## Discussion

This study investigates the effect of metabolic inhibition on I_Ca,L_ in frog ventricular myocytes. The key findings are as follows: first, metabolic inhibition caused a reduction of both basal and ISO stimulated I_Ca,L_, but ISO stimulated cells were more sensitive to metabolic inhibition. Second, the effect of metabolic inhibition on I_Ca,L_ was strongly attenuated by the inhibition of mitochondrial F_1_F_0_-ATP synthase and intracellular dialysis with non-hydrolysable ATP analogues. These findings indicate that the observed effects were not caused by impaired ATP production, but were rather due to profound ATP hydrolysis by F_1_F_0_-ATP synthase operating in the reverse mode. Finally, our results demonstrate that increased intracellular acidification is the primary cause of I_Ca,L_ inhibition.

After the onset of coronary occlusion, ischemic myocardium quickly loses its contractile function and electrical instability can lead to ventricular fibrillation and sudden cardiac death. Mitochondria are known to be a central player in myocardial ischemic injury [33]. Ischemia-induced loss of electrical potential across the inner mitochondrial membrane results in the suppression of oxidative phosphorylation, ATP depletion and opening of the mitochondrial permeability transition pore and mitochondrial apoptosis channels resulting in myocyte apoptosis and necrosis [33, 34]. It is also well documented that hypoxia leads to the reversible LTCC inhibition [35–38]. Interestingly, under hypoxic condition, increased sensitivity of I_Ca,L_ to β-adrenergic stimulation was observed in single cardiomyocytes [36, 37] as well as in isolated heart [39]. Similar sensitization of LTCCs to β-adrenergic stimulation was also reported when mitochondrial function was inhibited with myxothiazol or FCCP [37]. However, the cellular and molecular mechanisms underlying the effects of hypoxia on activity of LTCCs remain incompletely understood. An important role of protein kinase C in the modulation of the sensitivity of the channel to β-adrenergic receptor stimulation was suggested [36]. Additionally, it was reported that pre-exposure of the myocytes to extracellular hydrogen peroxide attenuated both the inhibition of basal I_Ca,L_ and the increase in sensitivity of I_Ca,L_ to β-adrenergic stimulation during hypoxia suggesting that hypoxia mediates changes in channel activity and sensitivity to adrenergic stimulation by a lowering in hydrogen peroxide levels [37]. As an alternative hypothesis, it was proposed that decrease of I_Ca,L_ during hypoxia is mediated by the binding of haem oxygenase to the CaM/CaMKII-specific motifs of LTCCS [38].

Pharmacologically induced metabolic inhibition was demonstrated to lead to development of action potential and Ca^2+^ alternans in the heart [40, 41] and to increase vulnerability to arrhythmias [42]. Metabolic inhibition-induced changes in excitation-contraction coupling are well documented [3, 4, 15, 43]. It was demonstrated that metabolic inhibition affects properties of intracellular Ca^2+^ release and decreases frequency of spontaneous Ca^2+^ waves [2, 3]. The reports on the changes in SR Ca^2+^ load during metabolic inhibition are rather inconsistent as some studies have reported no change or decrease in SR Ca^2+^ content [4, 5] while others demonstrated increase in SR Ca^2+^ load [2]. Such discrepancies in the results can be at least partially explained by the reported biphasic effect of metabolic inhibition on intracellular Ca^2+^ signaling, consisting of an initial inhibition followed by stimulation of SR Ca^2+^ release [3]. The primary focus of this study was to examine the effect of metabolic inhibition on the activity of LTCCs, which serve as a trigger for Ca^2+^-induced Ca^2+^-release in mammalian cardiomyocytes. Metabolic inhibition leads to the decrease in I_Ca,L_ amplitude [3, 4]. Single channels recordings have revealed that metabolic inhibition reduces open probability, mean open time and delays activation of LTCCs [15]. However, the mechanisms underlying the suppression of I_Ca,L_ are poorly understood. It was suggested that the depletion of ATP during metabolic inhibition might lead to a decrease in phosphorylation of the channels which is essential to maintain normal function of LTCCs [44, 45]. In contrary, others studies have demonstrated that intracellular perfusion with non-hydrolysable analogues of ATP can successfully prevent I_Ca,L_ suppression, arguing against the channel phosphorylation hypothesis and an allosteric modulation of the channel by ATP was proposed [46, 47]. The importance of ATP in sustaining normal I_Ca,L_ is also confirmed by our experiments where dialysis of the cells with ATP free solution caused a decrease in both basal and ISO stimulated current amplitudes and an increase in current rundown (Fig 3). However, this observation can hardly explain the fact that wide array of oxidative phosphorylation inhibitors induced suppression of LTCCs even when the cell was dialysed with a solution containing 3 mM of ATP (Fig 1C). In addition, when I_Ca,L_ was stimulated by intracellular application of cAMP, FCCP suppressed I_Ca,L_ in a manner similar to ISO stimulated cells (Fig 5), suggesting that the effect of FCCP on I_Ca,L_ was independent of the activity of β-adrenergic receptors or adenylyl cyclase. When the myocytes were dialyzed with a pipette solution containing both ATP and ATP-γ-S, which leads to irreversible protein thio-phosphorylation [29], FCCP still induced an inhibition of I_Ca,L_ (Fig 5). Therefore, it is reasonable to conclude that the effects of metabolic inhibition on I_Ca,L_ are not related to decreased phosphorylation of the channels.

While inhibition of mitochondrial F_1_F_0_-ATP-synthase alone had only minor effect on I_Ca,L_, we observed that it strongly attenuated the inhibitory effect of FCCP (Fig 4). Under normal conditions, F_1_F_0_-ATP-synthase is the site of ATP production. However, it was suggested that during ischemia or pharmacological inhibition of the oxidative phosphorylation F_1_F_0_-ATP-synthase, in an attempt to maintain the mitochondrial membrane potential, begins to run reverse becoming a powerful ATP consumer [26–28]. Furthermore, it was demonstrated that a block of F_1_F_0_-ATP-synthase might be beneficial during ischemic or anoxic conditions [28, 48]. To further confirm that ATP hydrolysis is necessary to suppress I_Ca,L_ during metabolic inhibition, the effect of FCCP was examined in cells dialysed with a pipette-filling solution containing only non-hydrolysable ATP analogues AMP-PCP and ATP-γ-S. Non-hydrolysable ATP analogues completely prevented inhibition of I_Ca,L_ during metabolic inhibition (Fig 6) clearly demonstrating that suppression of I_Ca,L_ during metabolic inhibition is associated with the intensive ATP hydrolysis by F_1_F_0_ ATP-synthase.

Metabolic inhibition as well as ischemia results in intracellular acidification [3, 19, 49, 50]. Intracellular acidosis in cardiomyocytes was demonstrated to regulate various processes including Ca^2+^-induced Ca^2+^-release [49], activity of ion channels [51], gap junctions [52] and phosphorylation levels [19]. Previous studies exploring the effect of acidosis on LTCC activity led to conflicting results, with studies showing either inhibition [53–55], no apparent effect [51, 56] or increase in I_Ca,L_ [57]. The reasons for such conflicting results remain unclear and might be related to the experimental approaches used to induce intracellular acidosis and/or to species differences. Furthermore, the changes in intracellular pH may not be uniform within the cell. For instance, the close colocalization of mitochondria with SR and plasma membrane [21, 22] may form restricted domains where metabolic inhibition causes higher changes in pH. In our study, acidosis was induced by metabolic inhibition and this resulted in an inhibition of I_Ca,L_ which was much more pronounced during β-adrenergic stimulation. Interestingly, in an earlier study performed in guinea-pig ventricular myocytes, the maximal response to isoprenaline, forskolin or intracellular cAMP was also found to be strongly reduced in myocytes pretreated with acidic external solution [58]. Our results that both high intracellular pH buffering capacity and intracellular dialysis of myocytes with alkaline solution can mitigate metabolic inhibition-induced suppression of β-adrenergic response of LTCCs (Fig 8) suggest an essential role of acidosis in this process.

## Conclusions

**Fig 9:**
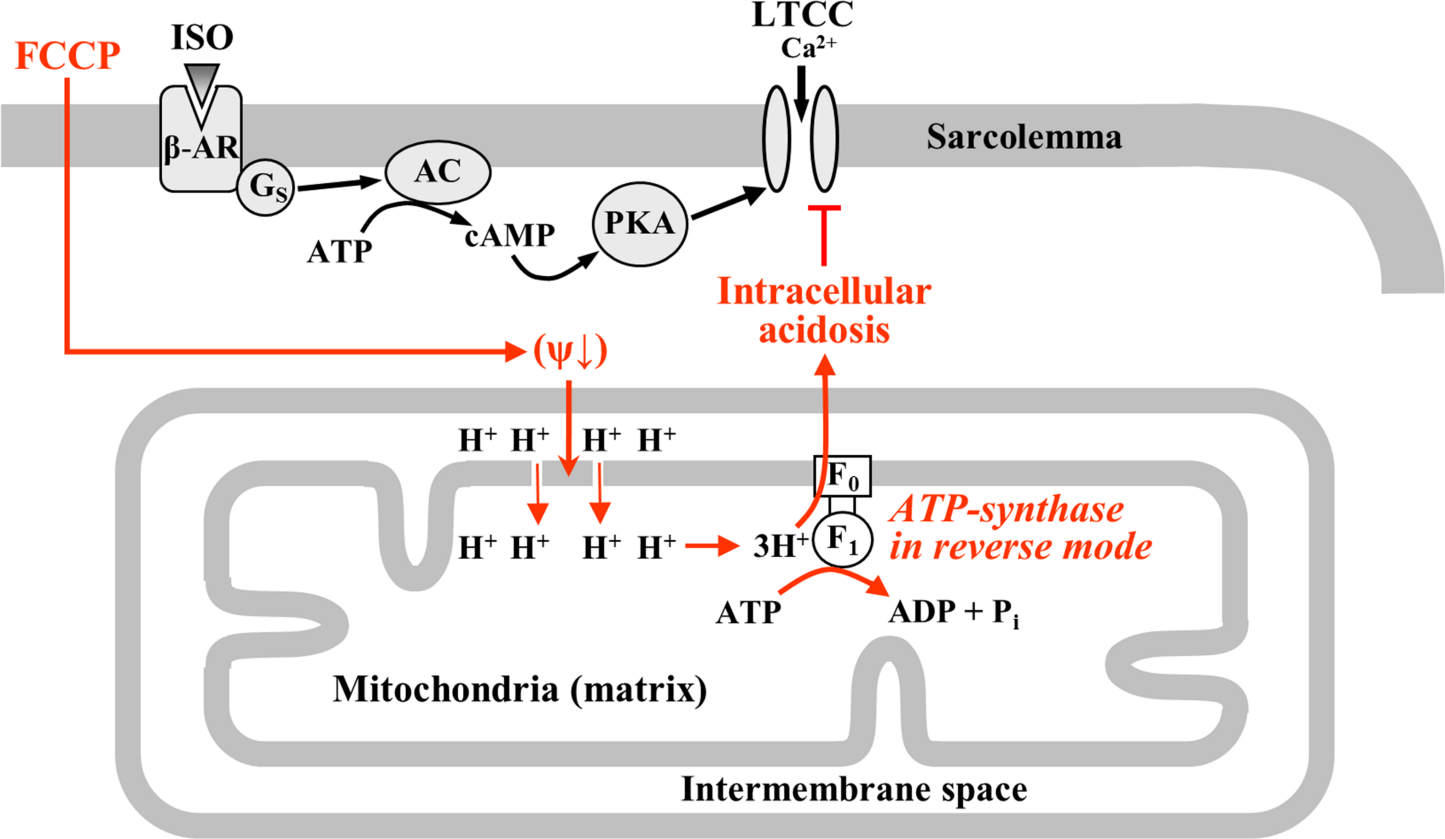
Schematic representation of the metabolic inhibition of cardiac I_Ca,L_. Metabolic inhibition leads to the depolarization of mitochondrial membrane potential. Under these conditions the mitochondrial F_1_F_0_-ATP synthase operates in the reverse-mode and intensive ATP hydrolysis results in intracellular acidification which in turn reduces I_Ca,L_.

Our data demonstrate that metabolic inhibition leads to excessive ATP hydrolysis by the mitochondrial F_1_F_0_-ATP-synthase operating in the reverse mode which results in intracellular acidosis (Fig 9). We suggest that acidosis plays a key role in the suppression of LTCC activity during metabolic inhibition, and this contributes to the mechanisms of ischemic injury.

## Supporting information

S1 Fig FCCP induced suppression of basal I_Ca,L_. (A) Dose dependent effect of FCCP on the peak amplitude of I_Ca,L_. (B) The mean peak amplitude of basal I_Ca,L_ in control and during exposure of myocytes to the increasing FCCP concentration (n = 5). * P<0.05 *vs*. control.
(PDF)

S2 Fig Effect of NaN_3_ on basal and ISO stimulated I_Ca,L_. A typical experiment representing effect of NaN_3_ on the peak amplitude of basal and ISO-stimulated I_Ca,L_. The current traces shown in the top panel were recorded at times indicated by the corresponding letters on the main graph.
(PDF)

S3 Fig Effect of FCCP on I_Ca,L_ in the cells dialyzed with ATP-γ-S. A time course of changes in ICa,L peak amplitude during the application of increasing FCCP concentrations to the ventricular myocytes dialyzed with internal solution containing 3 mM ATP and supplemented with 3 mM of ATP-γ-S. A transient application of ISO was used to induce irreversible thiophosphorylation of proteins. The current traces shown in the top panel were recorded at times indicated by the corresponding letters on the main graph.
(PDF)

S4 Fig Dialysis with non-hydrolysable ATP analogues prevents suppression of I_Ca,L_ by antimycin. An experiment demonstrating the lack of I_Ca,L_ suppression in the presence of 30 **μ**M of antimycin in a myocyte dialyzed with a pipette solution in which ATP was substituted with non-hydrolysable ATP analogues AMP-PCP (3 mM) and ATP-γ-S (0.5 mM). The current traces shown in the top panel were recorded at times indicated by the corresponding letters on the main graph.
(PDF)
