# Supplementary material for: Metabolic inhibition reduces cardiac L-type Ca2+ channel current due to acidification caused by ATP hydrolysis
Source: PLoS One. 2017 Aug 31;12(8):e0184246. doi: 10.1371/journal.pone.0184246 (PMC5578678; doi:10.1371/journal.pone.0184246)
Supplement: S1 Fig — (A) Dose dependent effect of FCCP on the peak amplitude of ICa,L. (B) The mean peak amplitude of basal ICa,L in control and during exposure of myocytes to the increasing FCCP concentration (n = 5). * P<0.05 vs. control. (PDF) [file pone.0184246.s001.pdf]

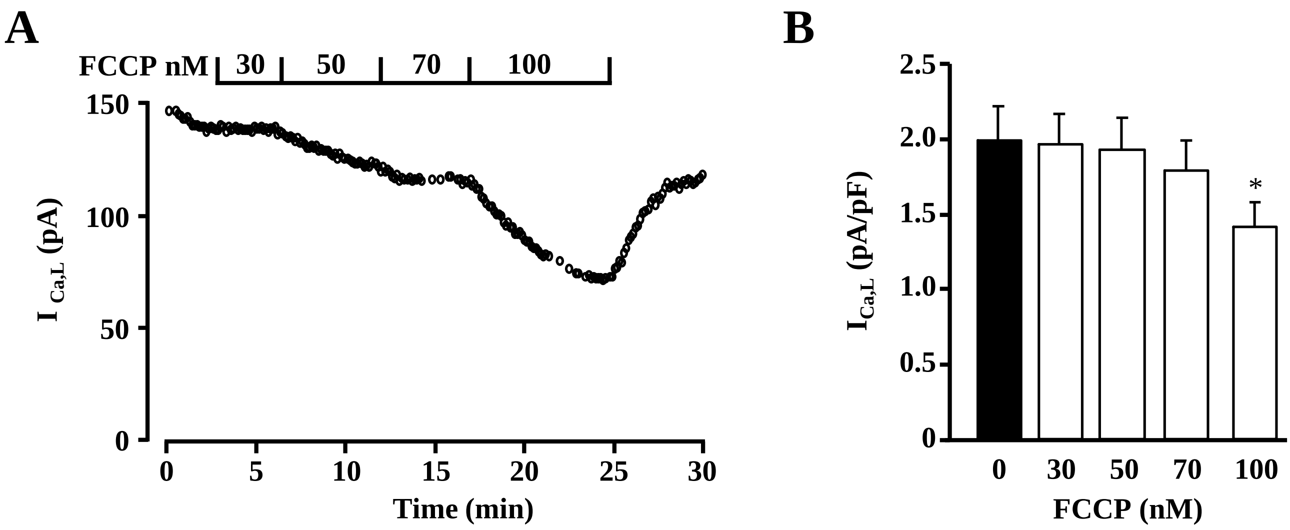

S1 Fig. FCCP induced suppression of basal $I_{Ca,L}$.

(A) Dose dependent effect of FCCP on the peak amplitude of $I_{Ca,L}$. (B) The mean peak amplitude of basal $I_{Ca,L}$ in control and during exposure of myocytes to the increasing FCCP concentration (n=5). ***** $P<0.05$ *vs.* control.
