# Supplementary material for: Metabolic inhibition reduces cardiac L-type Ca2+ channel current due to acidification caused by ATP hydrolysis
Source: PLoS One. 2017 Aug 31;12(8):e0184246. doi: 10.1371/journal.pone.0184246 (PMC5578678; doi:10.1371/journal.pone.0184246)
Supplement: S2 Fig — A typical experiment representing effect of NaN3 on the peak amplitude of basal and ISO-stimulated ICa,L. The current traces shown in the top panel were recorded at times indicated by the corresponding letters on the main graph. (PDF) [file pone.0184246.s002.pdf]

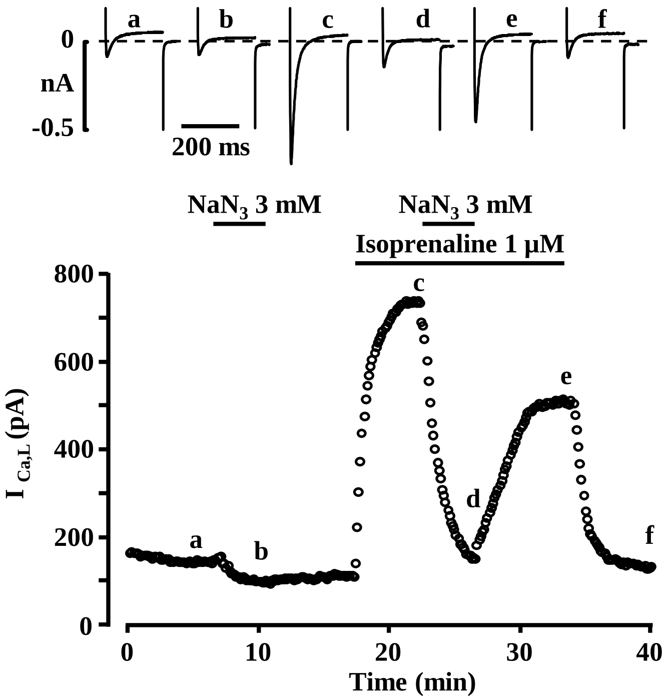

S2 Fig. Effect of NaN_3 on basal and ISO stimulated $I_{Ca,L}$.
A typical experiment representing effect of NaN_3 on the peak amplitude of basal and ISO-stimulated $I_{Ca,L}$. The current traces shown in the top panel were recorded at times indicated by the corresponding letters on the main graph.
