# Supplementary material for: Metabolic inhibition reduces cardiac L-type Ca2+ channel current due to acidification caused by ATP hydrolysis
Source: PLoS One. 2017 Aug 31;12(8):e0184246. doi: 10.1371/journal.pone.0184246 (PMC5578678; doi:10.1371/journal.pone.0184246)
Supplement: S3 Fig — A time course of changes in ICa,L peak amplitude during the application of increasing FCCP concentrations to the ventricular myocytes dialyzed with internal solution containing 3 mM ATP and supplemented with 3 mM of ATP-γ-S. A transient application of ISO was used to induce irreversible thiophosphorylation of proteins. The current traces shown in the top panel were recorded at times indicated by the corresponding letters on the main graph. (PDF) [file pone.0184246.s003.pdf]

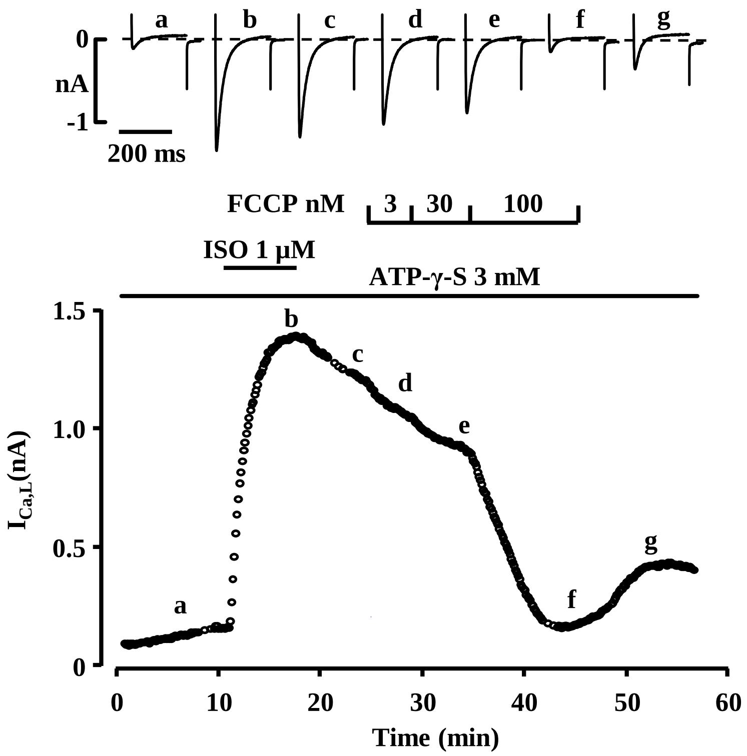

S3 Fig. Effect of FCCP on $I_{Ca,L}$ in the cells dialyzed with ATP-γ-S.

A time course of changes in $I_{Ca,L}$ peak amplitude during the application of increasing FCCP concentrations to the ventricular myocytes dialyzed with internal solution containing 3 mM ATP and supplemented with 3 mM of ATP-γ-S. A transient application of ISO was used to induce irreversible thiophosphorylation of proteins. The current traces shown in the top panel were recorded at times indicated by the corresponding letters on the main graph.
