# Supplementary material for: Metabolic inhibition reduces cardiac L-type Ca2+ channel current due to acidification caused by ATP hydrolysis
Source: PLoS One. 2017 Aug 31;12(8):e0184246. doi: 10.1371/journal.pone.0184246 (PMC5578678; doi:10.1371/journal.pone.0184246)
Supplement: S4 Fig — An experiment demonstrating the lack of ICa,L suppression in the presence of 30 μM of antimycin in a myocyte dialyzed with a pipette solution in which ATP was substituted with non-hydrolysable ATP analogues AMP-PCP (3 mM) and ATP-γ-S (0.5 mM). The current traces shown in the top panel were recorded at times indicated by the corresponding letters on the main graph. (PDF) [file pone.0184246.s004.pdf]

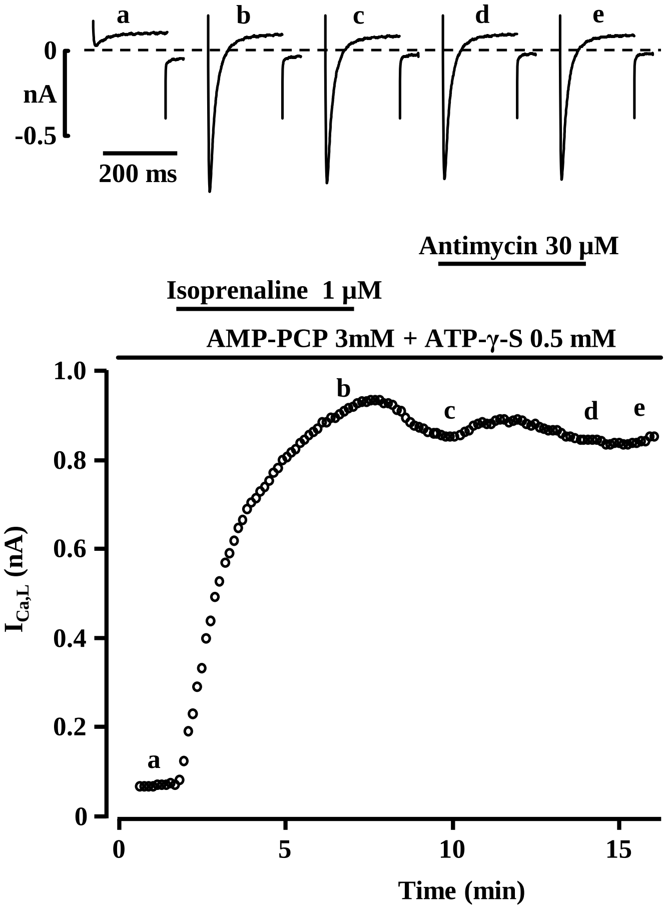

S4 Fig. Dialysis with non-hydrolysable ATP analogues prevents suppression of $I_{Ca,L}$ by antimycin.

An experiment demonstrating the lack of $I_{Ca,L}$ suppression in the presence of 30 µM of antimycin in a myocyte dialyzed with a pipette solution in which ATP was substituted with non-hydrolysable ATP analogues AMP-PCP (3 mM) and ATP-γ-S (0.5 mM). The current traces shown in the top panel were recorded at times indicated by the corresponding letters on the main graph.
